# Bioinformatics Screening of Potential Biomarkers from mRNA Expression Profiles to Discover Drug Targets and Agents for Cervical Cancer

**DOI:** 10.3390/ijms23073968

**Published:** 2022-04-02

**Authors:** Md. Selim Reza, Md. Harun-Or-Roshid, Md. Ariful Islam, Md. Alim Hossen, Md. Tofazzal Hossain, Shengzhong Feng, Wenhui Xi, Md. Nurul Haque Mollah, Yanjie Wei

**Affiliations:** 1 Centre for High Performance Computing, Joint Engineering Research Center for Health Big Data Intelligent Analysis Technology, Shenzhen Institute of Advanced Technology, Chinese Academy of Sciences, Shenzhen 518055, China; selim@siat.ac.cn (M.S.R.); tofazzal@siat.ac.cn (M.T.H.); sz.feng@siat.ac.cn (S.F.); wh.xi@siat.ac.cn (W.X.); 2 School of Computer Science and Technology, University of Chinese Academy of Sciences, Beijing 100049, China; 3 Bioinformatics Lab, Department of Statistics, University of Rajshahi, Rajshahi 6205, Bangladesh; harun.stat.ru@gmail.com (M.H.-O.-R.); ariful.stat.bio@gmail.com (M.A.I.); md.alimhossen@yahoo.com (M.A.H.)

**Keywords:** cervical cancer, mRNA expression profiles, key genes, candidate drugs, integrated bioinformatics analysis

## Abstract

Bioinformatics analysis has been playing a vital role in identifying potential genomic biomarkers more accurately from an enormous number of candidates by reducing time and cost compared to the wet-lab-based experimental procedures for disease diagnosis, prognosis, and therapies. Cervical cancer (CC) is one of the most malignant diseases seen in women worldwide. This study aimed at identifying potential key genes (KGs), highlighting their functions, signaling pathways, and candidate drugs for CC diagnosis and targeting therapies. Four publicly available microarray datasets of CC were analyzed for identifying differentially expressed genes (DEGs) by the LIMMA approach through GEO2R online tool. We identified 116 common DEGs (cDEGs) that were utilized to identify seven KGs (AURKA, BRCA1, CCNB1, CDK1, MCM2, NCAPG2, and TOP2A) by the protein–protein interaction (PPI) network analysis. The GO functional and KEGG pathway enrichment analyses of KGs revealed some important functions and signaling pathways that were significantly associated with CC infections. The interaction network analysis identified four TFs proteins and two miRNAs as the key transcriptional and post-transcriptional regulators of KGs. Considering seven KGs-based proteins, four key TFs proteins, and already published top-ranked seven KGs-based proteins (where five KGs were common with our proposed seven KGs) as drug target receptors, we performed their docking analysis with the 80 meta-drug agents that were already published by different reputed journals as CC drugs. We found Paclitaxel, Vinorelbine, Vincristine, Docetaxel, Everolimus, Temsirolimus, and Cabazitaxel as the top-ranked seven candidate drugs. Finally, we investigated the binding stability of the top-ranked three drugs (Paclitaxel, Vincristine, Vinorelbine) by using 100 ns MD-based MM-PBSA simulations with the three top-ranked proposed receptors (AURKA, CDK1, TOP2A) and observed their stable performance. Therefore, the proposed drugs might play a vital role in the treatment against CC.

## 1. Introduction

Cervical cancer (CC) is a type of malignancy that arises from the cervix (lower part of the uterus) and is characterized by expulsion and irregular bleeding in the vagina, pelvic pain, and pain during sexual intercourse [1]. It has been reported that the human papillomavirus (HPV) infection causes CC in almost all cases [2]. Currently, CC is placed as the fourth most common type of malignancy among females with a high mortality rate worldwide and the second most prevalent cancer among females in middle- and low-income countries (MLICs) [3,4]. According to the 2018 Globocan report, about 569,847 new CC cases with 311,365 mortalities are identified annually [3]. About 14,065 females are diagnosed with CC every year in the United States, causing 5266 deaths [5]. In China, CC is considered the eighth most common cancer in women but, surprisingly, the second most common cancer in women between 15 and 44 years old. About 43% of CC patients are <45 years of age, and 20–28% are <40 years of age [6]. However, cervical cancer has been relatively well controlled for several decades in many high-income countries due to the cervical screening initiatives and effective cancer treatment services, but it remains the most prevalent cause of cancer-related mortality among women in MLICs [4,7,8]. There is still debate about the precise molecular pathways between chronic high-risk HPV infection and the CC pathological phase [9]. Growing research has shown that the irregular expression of multiple genes is convoluted in the pathogenesis of CC [10]. Since tumorigenesis involves various complex genetic and epigenetic events, including the overexpression of oncogenes or the inactivation of suppressor genes [11], the revolution of dysregulated genes in oncogenic pathways might highlight the molecular events underlying tumor formation, which helps insight into CC treatment strategies. Therefore, it is crucial to elucidate the potential molecular mechanisms underlying CC to offer novel therapeutic targets and prognostic biomarkers of CC [12,13].

**Figure 1:**
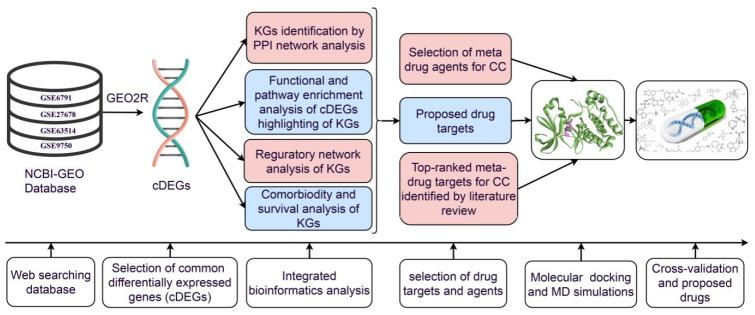
The pipeline of this study.

However, *de novo* (new) drug discovery is a tremendously challenging, time-consuming, and expensive task due to several steps involved in this process, from the target-based drug selection to clinical validation. Drug repurposing (DR) is a promising approach to overcome many of those obstacles in discovering and developing new drugs by exploring the new therapeutic applications of approved drugs that are established for different diseases [14]. It is considered as a supporting process to the conventional drug discovery. Exploring more suitable repurposable drugs for a new disease requires identifying appropriate target proteins associated with the disease. Hub genes/study genes mediated proteins have been considered as the key drug target receptors. Transcriptomics analysis is a widely used popular approach to explore genomic biomarkers [15,16,17,18,19,20,21]. By this time, several authors have suggested several sets of hub genes/study genes to explore molecular mechanisms and pathogenetic processes of CC [22,23,24,25]. CDK1 and TOP2A might play a critical role in controlling the genetic network related to cervical cancer incidence, progression, and metastasis [17,26,27]. Among them, some authors suggested candidate drugs for the treatment against CC [16,28]. Nevertheless, none of them have investigated the resistance of their suggested drugs against the independent receptors proposed by others. A question may be raised, namely, how can a drug be effective globally for all people around the world. Therefore, our main objectives are (i) computational identification of genomic biomarkers (drug targets) for CC, highlighting their functions, pathways, and regulatory factors, (ii) exploring proposed genomic biomarkers-guided candidate drugs for the treatment against CC, and (iii) in silico validation of the proposed drugs against the state-of-the-art alternative top-ranked independent receptors proposed by others. The workflow of the present research is displayed in Figure 1.

## 2. Results

### 2.1. Identification of cDEGs

**Figure 2:**
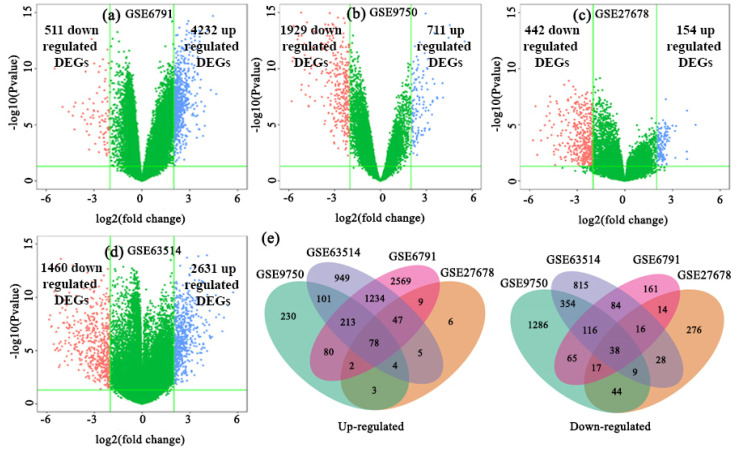
Screening of the overlapping DEGs among GSE6791, GSE27678, GSE63514, and GSE9750 datasets. The volcano plots of DEGs in (**a**) GSE6791, (**b**) GSE9750, (**c**) GSE27678, and (**d**) GSE63514; red dots and blue dots represented the significantly down-regulated and up-regulated DEGs, respectively. (**e**) Common up-regulated and down-regulated differentially expressed genes from CC visualized through a Venn diagram. Seventy-eight genes were founded common- up-regulated and thirty-eight genes were founded common down-regulated in CC patients.

The datasets GSE6791, GSE27678, GSE63514, and GSE9750 were analyzed to identify DEGs between CC infections and control samples, and the DEGs in each dataset were presented using the volcano plots (Figure 2a–d), where blue and red dots represented the up-regulated and down-regulated genes, respectively. In GSE6791, a total of 4743 DEGs with 4232 up-regulated and 511 down-regulated genes; in GSE27678, a total of 596 DEGs with 154 up-regulated and 442 down-regulated genes; in GSE63514, a total of 4091 DEGs with 2631 up-regulated and 1460 down-regulated genes; in GSE9750, a total of 2640 DEGs with 711 up-regulated and 1929 down-regulated genes were identified by GEO2R online tool with |logFC| > 1.0 and adjusted *p*-value < 0.05. Then, we found 78 up-regulated cDEGs and 38 down-regulated cDEGs for CC patients (see Figure 2e; Table S3).

### 2.2. PPI Network Analysis of cDEGs for Identification of KGs

**Figure 3:**
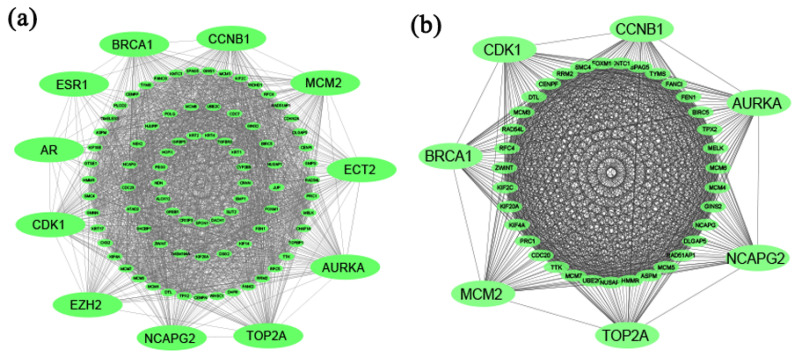
(**a**) Protein–protein interaction network for common differentially expressed genes of CC, and edges specify the interconnection in the middle of two genes. The analyzed network holds 92 nodes and 1790 edges. Surrounding nodes (AR, AURKA, BRCA1, CCNB1, CDK1, ECT2, ESR1, EZH2, MCM2, NCAPG2, and TOP2A) represented the hub genes. (**b**) Module analysis network obtained from MCODE analysis. Surrounding nodes (AURKA, BRCA1, CCNB1, CDK1, MCM2, NCAPG2, and TOP2A) were found to be common across 11 hub genes, so we considered these 7 genes as the key genes. The network represents highly interconnected regions of the PPI network. The network holds 42 nodes and 850 edges.

The PPI network of cDEGs was constructed using STRING database (Figure 3a), which contained 92 nodes and 1790 edges. We selected top-ranked eleven (11) cHubGs {AR, AURKA, BRCA1, CCNB1, CDK1, ECT2, ESR1, EZH2, MCM2, NCAPG2, and TOP2A}, applying four topological measures in the PPI network. Then, using MCODE, clusters were selected from the PPI network. It was shown that the most significant cluster had 42 nodes and 850 edges. MCODE analysis demonstrated that the most significant cluster contained the seven hub genes {AURKA, BRCA1, CCNB1, CDK1, MCM2, NCAPG2, and TOP2A} (see Figure 3b). So, we considered these seven key genes (KGs) for further analysis.

### 2.3. The Regulatory Network Analysis of KGs

**Figure 4:**
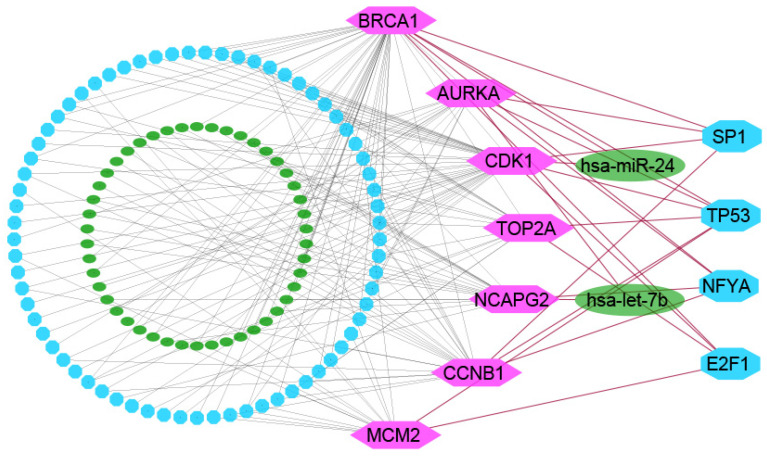
TFs-genes–miRNAs interaction network with KGs. The highlighted pink-color nodes represent the key genes, cyan-color nodes represent the TFs, and other dark-green-color nodes represent miRNAs. The network consists of 131 nodes and 175 edges.

The network analysis of KGs with TFs detected top-ranked four significant TFs (SP1, TP53, NFYA, and E2F1) as the key transcriptional regulatory factors for KGs (see Figure 4). We found TP53 as key TFs for six KGs (AURKA, BRCA1, CCNB1, CDK1, MCM2, and TOP2A), SP1 for four KGs (AURKA, BRCA1, CCNB1, and CDK1), NFYA for four KGs (AURKA, BRCA1, CCNB1, and NCAPG2), and E2F1 for four KGs (AURKA, CDK1, MCM2, and TOP2A). Similarly, the network analysis of KGs with miRNAs identified top-ranked two significant miRNAs, denoted as hsa-mir-24 and hsa-let-7b, that were considered as the key post-transcriptional regulatory factors for KGs sets {AURKA, BRCA1, and CDK1}, {CCNB1, CDK1, and NCAPG2}, respectively (see Figure 4).

### 2.4. GO Functions and KEGG Pathway Enrichment Analysis of cDEGs Highlighting KGs

Table 1 displayed the top five significantly enriched GO terms and KEGG pathways by involving KGs of CC diseases that are also supported by the literature review [17,19,23,29,30,31,32,33,34,35,36]. The top five GO terms of the biological process, including DNA replication, cell division, G1/S transition of mitotic cell cycle, DNA replication initiation, mitotic nuclear division, were significantly enriched by the KGs sets {BRCA1, CDK1, MCM2}, {NCAPG2, AURKA, CCNB1, CDK1}, {CDK1, MCM2}, {MCM2}, and {NCAPG2, AURKA, CDK1}, respectively. The MFs GO terms ATP binding, protein binding, DNA helicase activity, chromatin binding, and DNA binding were significantly enriched by the KGs sets {TOP2A, AURKA, CDK1, MCM2}, {TOP2A, NCAPG2, BRCA1, MCM2, AURKA, CCNB1, CDK1}, {MCM2}, {TOP2A, CDK1}, and {TOP2A, BRCA1, MCM2}, respectively. The cellular components GO terms nucleoplasm, midbody, MCM complex, nucleus, and spindle were significantly enriched by the KGs sets {TOP2A, NCAPG2, BRCA1, MCM2, AURKA, CCNB1, CDK1}, {AURKA, CDK1}, {MCM2}, {TOP2A, NCAPG2, BRCA1, MCM2, AURKA, CCNB1, CDK1}, and {AURKA}, respectively. We observed that KEGG pathway including DNA replication, cell cycle, p53 signaling pathway, Oocyte meiosis, and Fanconianemia were significantly enriched by the KGs sets {MCM2}, {CCNB1, CDK1, MCM2}, {CCNB1, CDK1}, {CCNB1, CDK1, AURKA}, and {BRCA1}, respectively. The other significantly enriched GO terms and KEGG pathways of cDEGs were given in Table S4.

### 2.5. Survival Analysis with KGs

**Figure 5:**
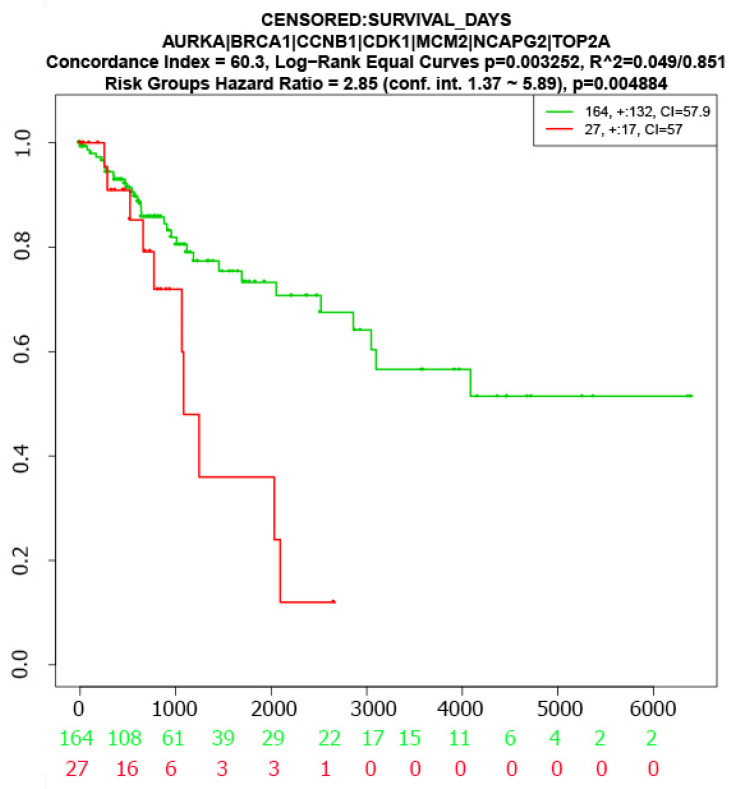
The multivariate survival curves of CC patients based on KGs.

The log-rank test was used to test the significant difference between two survival curves corresponding to low- and high-risk groups based on the seven KGs (AURKA, BRCA1, CCNB1, CDK1, MCM2, NCAPG2, and TOP2A). We observed a significant difference between the two survival probability curves (Figure 5), which indicates that the proposed KGs have strong prognostic power in detecting CC.

### 2.6. Drug Repurposing by Molecular Docking

To explore candidate drugs by molecular docking simulation, we considered m = 11 drug target proteins (receptors) corresponding to our proposed seven KGs and their regulatory four key TFs and 80 meta-drug agents (ligands) as mentioned in Section 4.3. We downloaded the 3D structure of our proposed 11 receptors (AURKA, BRCA1, CCNB1, CDK1, MCM2, TOP2A, SP1, TP53, NFYA, E2F1) from the Protein Data Bank (PDB) [37] with source codes 1mq4, 1n5o, 2b9r, 4y72, 4uuz, 1zxm, 1sp1, 1aie, 6qmq, 2aze, respectively. The 3D structure of the NCAPG2 target protein was downloaded from AlphaFold source using UniProt [38] ID of Q86XI2. The 3D structures of 80 drugs (see Table S1) were downloaded from the PubChem database [39] as mentioned previously.

**Figure 6:**
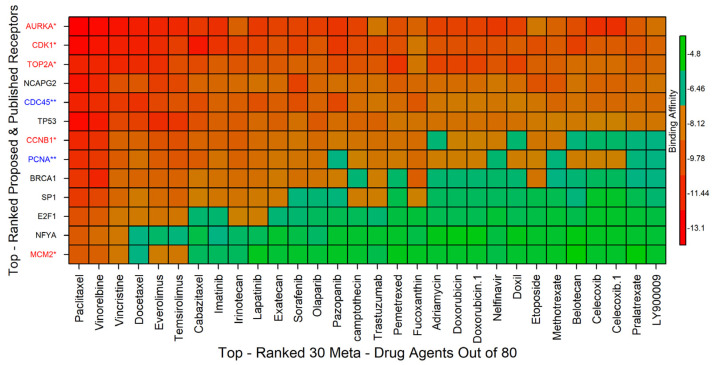
Image of binding affinities based on the top-ordered 30 meta-drug agents out of 80 against the ordered 13 receptors, where red colors indicated the strong binding affinities. The red color with a single star (*) indicated common receptors (published and proposed), the blue color with a double star (**) denoted only published receptors, and the receptors with the black color indicated only proposed receptors.

On the other hand, we reviewed 52 published articles associated with CC infections that provided transcriptome-guided hub proteins (genomic biomarkers) for cross-validation of the proposed key genes and the candidate drug agents. There were 255 hub genes reported in those 52 articles, with 7 hub proteins (AURKA, PCNA, CCNB1, CDC45, MCM2, TOP2A, CDK1) appearing in at least 5 articles (Table S2) [15,16,17,18,19,20,21,23,24,25,26,27,40,41,42,43,44,45,46,47,48,49,50,51]. Five (AURKA, CCNB1, MCM2, TOP2A, and CDK1) of the seven reported hub proteins were found to be similar to our suggested seven KGs. We downloaded the 3D structure of the published remaining receptors (CDC45, PCNA) from PDB with source codes 5dgo, 1u76, respectively. Then, molecular docking was carried out between total *m* = 13 receptors (proposed and published) and *n* = 80 meta-drug agents to calculate the binding affinity scores (kcal/mol) for each pair of receptors and agents. Then, we ordered the target receptors in descending order of row sums of the binding affinity matrix *A* = (*A*_ij_) and drug agents, according to the column sums, to select a few drug agents as the candidate drugs (see Figure 6). Thus, we selected top-ranked three drug agents (Paclitaxel, Vinorelbine, Vincristine) as candidate drugs with binding affinity scores −7.5 kcal/mol ≤ against the 13 receptors.

**Figure 7:**
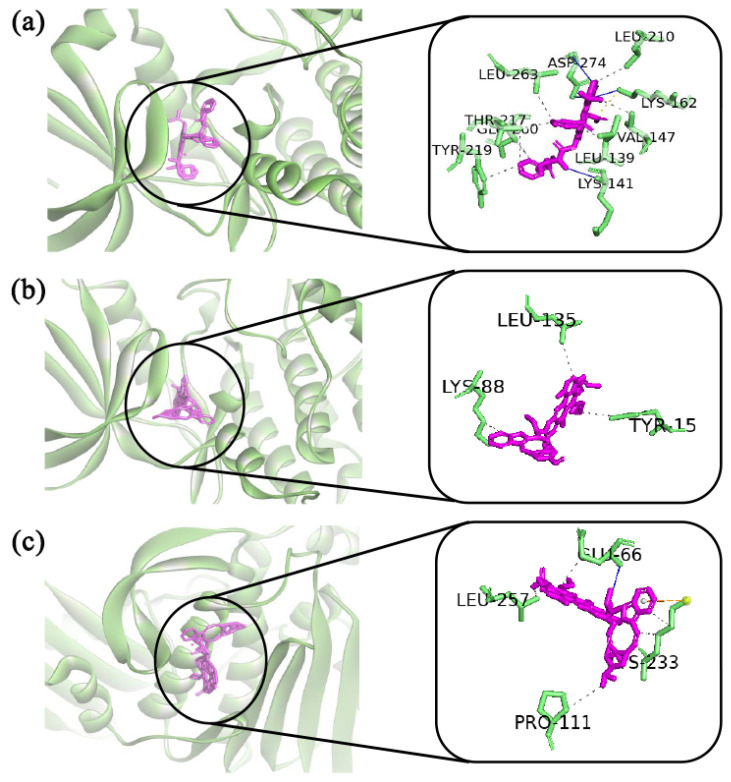
The top-ranked three complexes obtained from molecular docking study and their two-dimensional chemical interactions showed in the right side. The figure is generated by using discovery studio visualizers, PLIP and PyMol software. Complexes: (**a**) indicated AURKA_paclitaxel, (**b**) indicated CDK1_pinorelbine, and (**c**) indicated TOP2A_pincristine.

The docked complexes of the top three virtual hits from AutoDock-Vina docking were further considered for protein–ligand interaction profiling. As shown in Figure 7a, AURKA_paclitaxel complex showed three hydrogen bonds with lys141, lys162, asp274 residues. Although the ligand formed major hydrophobic interactions with leu139, val147, leu210, thr217, tyr219, glu260, leu263 residues, and lys162 residue showed additional salt bridges interactions with the ligand. On the other hand, CDK1_vinorelbine (Figure 7b) formed hydrophobic interactions with tyr15, lys88, leu135 residues. In the case of the TOP2A_vincristine complex, vincristine showed hydrogen bonding to the active site, where the interaction was maintained by glu66 residue. Vincristine also formed hydrophobic interactions with glu66, pro111, lys233, leu257 residues. Furthermore, vincristine also formed π-cation interactions with lys233 residue (see Figure 7c).

### 2.7. MD Simulations

Among the proposed candidate drugs, Paclitaxel, Vinorelbine, and Vincristine were the top-ranked three candidate drugs (Figure 6). Therefore, these top three drug agents were selected for their stability analysis through 100 ns MD-based MM-PBSA simulations.

**Figure 8:**
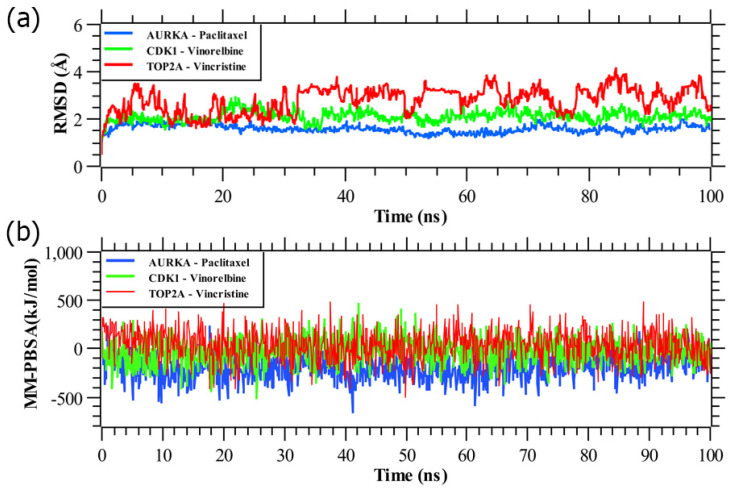
(**a**) Time evolution of root mean square deviations (RMSDs) of backbone atoms (C, Cα, and N) for protein for each docked complex. (**b**) Binding free energy (in kJ mol^−1^) of each snapshot was calculated by molecular mechanics Poisson–Boltzmann surface area (MM-PBSA) analysis, representing the change in binding stability of each complex during simulations; positive values indicate better binding. Complexes: blue AURKA, green CDK1, and red TOP2A.

From Figure 8, we observed that all the three systems were significantly stable between the variations of moving and initial drug–target complexes. We calculated their RMSD (root mean square deviation). Figure 8a represented the RMSD corresponding to the proposed receptors (AURKA, CDK1, TOP2A). All the systems projected the RMSD around 1 Å to 2.5 Å, except TOP2A complex, which shows the RMSD around 2 Å to 3.7 Å. The average RMSD for AURKA, CDK1, TOP2A complexes were 1.59 Å, 2.11 Å, and 2.80 Å, respectively. CDK1 complex showed slight fluctuation in around 20,000 ps to 28,000 ps and was stabilized in the remaining simulation. As can be seen from the plot, AURKA showed a more rigid conformation than the other proteins, also achieved equilibrium at 3 ns, and remained stable afterward. In contrast, TOP2A showed a dramatic increase in flexibility, with RMSD values rising gradually from 2 Å to 3.5 Å over time. Here, we calculated the MM-PBSA binding energy for three drug agents as mentioned previously, Figure 8b represented the binding energy with the top-ranked three proposed potential biomarkers (AURKA, CDK1, TOP2A). On an average, AURKA, CDK1, TOP2A complexes produced binding energies −192.65 kJ/mol, −26.36 kJ/mol, and 41.56 kJ/mol, respectively.

## 3. Discussion

CC is the second most common type of malignant tumor in females with a 5-year survival rate of only about 52%. Thus, more research is required to discover potential biomarkers and candidate drugs for improving the 5-year survival rate and reducing the mortality rate of CC patients [16].

To investigate the genetic influence of CC infections, we identified 116 shared cDEGs. Among them, we detected seven cDEGs as KGs highlighting their functions, pathways, regulatory factors, and candidate drugs. The literature review suggested that AURKA was an enhanced potential target for cervical cancer treatment [15,25,27,43,45]. BRCA1 enhanced the sensitivity of cervical squamous cell carcinoma (CSCC) patients to cisplatin-based CCRT by up-regulating STAT1 to activate the JAK/STAT pathway [50]. CCNB1 played vital roles in the progression of CC through different signaling pathways [17,18,26,46,51]. CDK1 contributed to the occurrence and development of CSCC [18,23,25,26,27,40,41,44,46]. MCM2 involved in the carcinogenesis of cervical cancer [16,19,20,21,23,27,42]. TOP2A is regarded as a biomarker for the improved diagnosis of CC [16,17,19,23,24,26,27,41,43,45]. We also investigated the multivariate survival analysis of CC patients with KGs and found a substantial difference between two survival probability curves, indicating that the suggested KGs have a better prognostic capacity for CC detection. Four transcriptional (TFs) and two post-transcriptional (miRNAs) regulatory factors of KGs were introduced in Section 2.3. The four TFs proteins (SP1, TP53, NFYA, E2F1) were further utilized as the drug target receptors. Previous studies also supported that SP1 is an important biomarker for CC [52]. Khan M.A et al. revealed that TP53 has an association with cervical cancer pathogenesis [53]. NFYA promoted cell proliferation and tumorigenic properties by transcriptional activation of SOX2 in cervical cancer [54]. The E2F1 protein is considered as a potential biomarker for CC [55].

To investigate the common pathogenetic processes of KGs, we selected the top five common GO terms for each of BPs, MFs and cellular components, and KEGG pathways that were significantly associated with cervical cancer disease through the cDEGs, including KGs. Among them, the association of the top five common BPs (DNA replication, cell division, G1/S transition of mitotic cell cycle, DNA replication initiation, mitotic nuclear division) with CC was supported by some other individual studies [17,19,30]. The top five common MFs (ATP binding, protein binding, DNA helicase activity, chromatin binding, and DNA binding) that were significantly associated with CC disease also received support from some individual studies [17,19,31,32,33]. Similarly, the association of the top four cellular components (nucleoplasm, midbody, MCM complex, nucleus, and spindle) with CC disease was supported by the literature review [19,34,35,36]. We selected the top five significantly enriched common KEGG pathways (DNA replication, cell cycle, p53 signaling pathway, Oocyte meiosis, and Fanconianemia pathway) that were also reported by some other studies [17,23,29].

**Figure 9:**
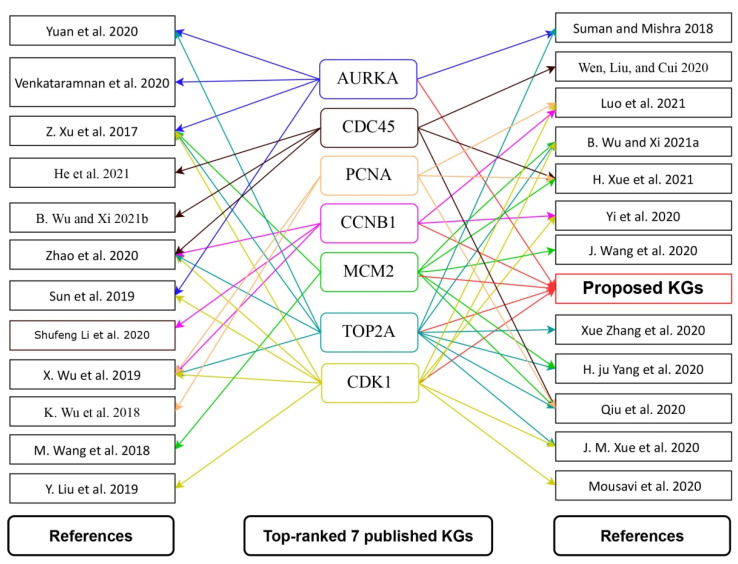
Top-ranked seven published hub genes, five of them identical to our proposed KGs for CC, where the first and third columns indicated different articles, and the second column indicated the top-ranked hub genes, where, red-color connected networks indicated the proposed KGs, and different-color connected networks indicated the different top-ranked hub genes.

To explore effective candidate drugs for the treatment against CC disease, we considered top-ranked proposed receptors and their regulatory four key TFs proteins as the drug target receptors and performed their docking analysis with 80 meta-drug agents (see Table S1). For cross-validation, we considered seven published hub proteins, where five proteins were common with our proposed KGs (see Figure 9). On the other hand, five articles at a time explored four hub genes individually, two articles explored three hub genes individually, etc. Moreover, TOP2A gene supported by 11 articles, CDK1 gene was supported by 10 articles, MCM2 gene was supported by 7 articles, CDC45 gene was supported by 6 articles, and (AURKA, CCNB1, PCNA) genes were supported by 5 articles (see Figure 9). Then, we selected top-ranked three drugs as the candidate drugs, where the first three drugs showed strong binding affinities with all target proteins (Figure 6), where red * denoted published and proposed receptors, and blue ** denoted published receptors. Some other independent studies also recommended our suggested drugs, including Vincristine [56,57], Vinorelbine [57,58], and Paclitaxel [56,57,58,59,60], for the treatment against CC. Finally, we examined the stability of top-ranked three drugs (Vincristine, Vinorelbine, Paclitaxel) by using 100 ns MD-based MM-PBSA simulations for three top-ranked proposed receptors (AURKA, CDK1, TOP2A) and observed their stable performance according to the laws of physics [61,62]. Therefore, the proposed candidate drugs might play a vital role in the treatment against CC with comorbidities, since our proposed target proteins are also associated with several comorbidities. The present study emphasizes the further wet-lab experimental validation for both the proposed target proteins and candidate drugs.

## 4. Materials and Methods

### 4.1. Data Sources and Descriptions

We used both original data and meta-data to reach the goal of this study as described below.

### 4.2. Collection of Microarray Exploring Profiles for Genomic Biomarkers and Drug Target Receptors

We collected microarray profiles for cervical cancer (CC) disease from the National Center for Biotechnology Information (NCBI) Gene Expression Omnibus (GEO) website (http://www.ncbi.nlm.nih.gov/geo/, (accessed on 10 March 2022)). CC patients microarray datasets of GSE6791 [63], GSE27678 [64], and GSE63514 [65] were all based upon the GPL570 Platforms ((HG-U133_Plus_2) Affymetrix Human Genome U133 Plus 2.0 Array), which included 20 CC tissues and 8 normal cervical tissues, 28 CC tissues and 3 normal cervical tissues, and 28 CC tissues and 24 normal cervical tissues, respectively. The GSE9750 [66] dataset used the GPL96 Affymetrix Human Genome U133A Array platform and included 33 CC tissue samples that were primarily marked by HPV16 or HPV18 and 21 normal cervical samples.

### 4.3. Collection of Meta-Drug Agents for Exploring Candidate Drugs

We collected host-transcriptome-guided 80 meta-drug agents by the literature review of CC disease (see Table S1) for exploring candidate drugs. Thus, we considered 80 drug agents to explore candidate drugs by molecular docking with our proposed receptors.

### 4.4. Collection of Independent Meta-Receptors for Cross-Validation with the Proposed Drugs

To select the top-ranked hub genes (independent meta-receptors) associated with CC disease, we reviewed 52 published articles and selected the top-ranked 7 target proteins as the independent meta-receptors (see Table S2).

### 4.5. Identification of cDEGs for CC Patients

The identification of cDEGs was a key step of this study. To identify cDEGs, we identified DEGs for each of GSE6791, GSE27678, GSE63514, and GSE9750 datasets separately, by using linear models for microarray (LIMMA) approach through GEO2R online tool [67] with |logFC| > 1.0 and adjusted *p*-value < 0.05. The LIMMA approach calculates the *p*-value by using the modified t-statistics to test the significance of differential gene expressions between two conditions, and *p*-value is then adjusted by using the Benjamini–Hochberg procedure [68]. Finally, we selected the common DEGs by using four DEGs sets derived from four publicly available microarray datasets.

### 4.6. Construction of Protein–Protein Interaction (PPI) Network for Identification of KGs

The PPI network of cDEGs was constructed through the STRING online database (https://string-db.org/, (accessed on 10 March 2022)) [69]. To improve the quality of PPI network, we used the Cytoscape software [70]. The Cytoscape plugin cytoHubba was used to select the common Hub Genes (cHubGs) or common Hub Proteins (cHubPs) from PPI network [70,71]. The PPI network provides several nodes and edges, which indicate proteins and their interactions, respectively. A node with the largest number of significant interactions/connections/edges with other nodes is considered as the top-ranked cHubGs. The cHubGs were selected by using four topological analyses (Degree [72], BottleNeck [73], Betweenness [74], and Stress [75]) of the PPI network. Molecular Complex Detection (MCODE) (http://apps.cytoscape.org/apps/mcode, (accessed on 10 March 2022)) plugin of Cytoscape software was used to detect the most profound modules from the PPIs network. Highly interconnected portions were identified through MCODE clustering that assists the research in effective drug designing. For representing molecular complexes in the PPI network, MCODE was used by detecting the densely connected areas [76]. Then, we selected the important key genes (KGs) that were shared by both cHubGs and MCODE clustering genes.

### 4.7. Regulatory Network Analysis of KGs

To explore key transcriptional regulatory transcription factors (TFs) and post-transcriptional regulatory micro-RNAs (miRNAs) of KGs, we performed TFs–TGs and miRNAs–KGs interaction network analysis, respectively, by using the NetworkAnalyst web platform [77]. The TFs–KGs and miRNAs–KGs interaction networks were constructed by using the ENCODE (https://www.encodeproject.org/, (accessed on 10 March 2022)) [78] and RegNetwork repository [79] databases, respectively. The Cytoscape software [70] was used to improve the quality of networks. The key regulators were selected by using two topological analyses (Degree [72] and Betweenness [74]) of networks.

### 4.8. GO Terms and KEGG Pathway Enrichment Analysis of KGs

Gene ontology (GO) functional and Kyoto Encyclopedia of Genes and Genomes (KEGG) pathway enrichment/annotation/over-representation analysis [80,81] is a widely used approach to determine the significantly annotated/enriched/over-represented functions/classes/terms and pathways by the identified cDEGs/cHubGs. It is an important part for revealing the molecular mechanisms of actions and cellular roles of genes. The GO terms are categorized into Biological Process, Cellular Component, and Molecular Function [82]. We performed GO and KEGG enrichment analysis using DAVID web tool (https://david.ncifcrf.gov/tools.jsp, (accessed on 10 March 2022)) [83]. The significance level was set to *p*-value < 0.05.

### 4.9. Survival Analysis

To examine the prognostic performance of KGs in detecting CC, we performed a multivariate survival analysis of CC patients based on expressions of KGs by using *SurvExpress online* tool [84]. The significance level was set to *p*-value < 0.05.

### 4.10. Drug Repurposing by Molecular Docking Study

To propose in silico validated efficient candidate drugs for the treatment against CC, we employed a molecular docking study of our proposed receptor proteins with the drug agents. We considered our proposed KGs-based key proteins (KPs) and their regulatory key TFs proteins as the drug target proteins and 80 drug agents as mentioned earlier in Section 4.3 (see Table S1). The molecular docking study requires three-dimensional (3D) structures of both receptor proteins and drug agents/ligands. We downloaded the 3D structure of all targeted proteins from the Protein Data Bank (PDB) [37] and SWISS-MODEL [85]. The 3D structures of all drug agents were downloaded from PubChem database [39]. The 3D structures of the target proteins were visualized using Discovery Studio Visualizer 2019 [86], and protein chains that were not part of the gene were removed. PDB2PQR and H++ servers were utilized to assign the protonation state of target proteins [87,88]. All the missing hydrogen atoms were also appropriately added. The pKa for target proteins residues were investigated under the physical conditions of salinity = 0.15, internal dielectric = 10, pH = 7, and external dielectric = 80. Further, the protein was prepared for molecular docking by removing ligand heteroatoms and water molecules and by addition of polar hydrogens on AutoDock tools 1.5.7 (developed by ADT, The Scripps Research Institute, La Jolla, CA, USA) [89]. The drug agents/ligands were prepared for molecular docking simulation by setting the torsion tree and rotatable/non-rotatable bonds present in the ligand through AutoDock tools 1.5.7. Then, binding affinities between the target proteins and drug agents were calculated using AutoDock Vina [90]. The exhaustiveness parameter was set to 10. Discovery Studio Visualizer 2019 [86], PLIP [91], and PyMol [92] were used to analyze the docked complexes for surface complexes, types, and distances of non-covalent bonds. Let *A_ij_* denotes the binding affinity between *i*th target protein (*I* = 1, 2, …, *m*) and *j*th drug agent (*j* = 1, 2, …, *n*). Then, target proteins were ordered according to the descending order of row sums $\overset{n}{\underset{j=1}{\sum }}A_{ij}$, *j* = 1, 2, …, *m*, and drug agents were ordered according to the descending order of column sums $\overset{m}{\underset{i=1}{\sum }}A_{ij}$, *j* = 1, 2, …, *n*, to select the few top-ranking drug agents as the candidate drugs. Then, we validated the proposed repurposed drugs by molecular docking study with the top-listed receptor proteins associated with CC infections that were obtained by the literature review. To select the top-listed receptor proteins associated with CC infections, we reviewed 52 recently published articles and selected the top-ranked seven receptor proteins (see Table S2).

### 4.11. Molecular Dynamic (MD) Simulations

MD simulations were carried out by using YASARA Dynamics software [93] and the AMBER14 force field [94] to study the dynamic behavior of the top-ranked protein–ligand complexes. A total of six different systems were used to run MD simulations. The systems included the top three hits, AURKA_paclitaxel, CDK1_vinorelbine, and TOP2A_vincristine complexes corresponding to our proposed receptors.

For the complexes, the parameters of ligands were assigned through AutoSMILES [95] algorithms, where unknown organic molecules are parameterized fully automatically by the calculation of semi-empirical AM1 Mulliken point charges with the COSMO solvation model, assigning of AM1BCC [96] atom and bond types, and also assigning general AMBER force field (GAFF) [97] atom types and the remaining force field parameters. Before the simulation, the protein–ligand complexes hydrogen bonding network was optimized and solvated by a TIP3P [98] water model in a simulation cell. Periodic boundary conditions were maintained with a solvent density of 0.997 g L^−1^. Titratable amino acids in the protein complex were subjected to pKa calculation during solvation. The initial energy minimization process of each simulation system, consisting of 55,410 ± 10, 72,287 ± 10, and 96,252 ± 10 atoms for AURKA_paclitaxel, CDK1_vinorelbine, and TOP2A_vincristine complexes, was performed by a simulated annealing method, respectively, using the steepest gradient approach (5000 cycles). Each simulation was run with a multiple-time-step algorithm [99], using a time-step interval of 2.50 fs under physiological conditions (298 K, pH 7.4, 0.9% NaCl) [100]. All bond lengths were constrained using the linear constraint solver (LINCS) [101] algorithm, and SETTLE [102] was used for water molecules. Long-range electrostatic interactions were described by the PME [103] methods, and finally, 100 ns MD simulation was accomplished at Berendsen thermostat [104] and constant pressure. The trajectories were recorded every 250 ps for further analysis, and subsequent analysis was implemented by the default script of YASARA [105] macro and SciDAVis software available at http://scidavis.sourceforge.net/, (accessed on 10 Mar 2021). All snapshots were then subjected to YASARA software’s MM-Poisson–Boltzmann surface area (MM-PBSA) binding free energy calculation using the formula below [106]:

(1) $$\begin{array}{cc} \mathrm{Binding}\;\mathrm{free}\;\mathrm{Energy} & \\ = & E_{\mathrm{potReceptor}}+E_{\mathrm{solvReceptor}}+{\;E}_{\mathrm{potLigand}}+{\;E}_{\mathrm{solvLigand}}\\ - & E_{\mathrm{potComplex}}-{\;E}_{\mathrm{solvComplex}} \end{array}$$

Here, MM-PBSA binding energy was calculated using YASARA built-in macros using AMBER 14 as a force field, with larger positive energies indicating better binding [107].

## 5. Conclusions

The present study utilized various well-established bioinformatics tools to reveal KGs, highlighting their regulatory factors and dysregulated molecular functions and pathways that were responsible for CC progression. The five KGs were common between our proposed seven KGs and the top-ranked seven KGs published by others, which indicated that our proposed KGs received more support by the literature review compared to any other individual studies. Finally, we suggested potential drugs, such as vincristine, vinorelbine, paclitaxel, and investigated their stability performance by using 100 ns MD-based MM-PBSA simulations for three top-ranked proposed receptors (AURKA, CDK1, TOP2A), also observing their stable performance. Thus, the proposed molecular biomarkers and repurposing candidate drugs presented in this study have merit for diagnosis and therapies of CC disease.

## Figures and Tables

**Table 1:** The top five significantly (*p*-value < 0.001) enriched GO functions and KEGG pathways by cDEGs involving KGs with CC diseases.

	**GO ID**	**GO Term**	**cDEGs** **(Counts)**	***p*-Value**	**Associated KGs**
**Biological Process** **(BPs)**	GO:0006260	DNA replication	18	1.58 × 10^−16^	BRCA1, CDK1, MCM2
	GO:0051301	Cell division	22	7.94 × 10^−15^	NCAPG2, AURKA, CCNB1, CDK1
	GO:0000082	G1/S transition of mitotic cell cycle	12	5.61 × 10^−11^	CDK1, MCM2
	GO:0006270	DNA replication initiation	8	1.18 × 10^−9^	MCM2
	GO:0007067	Mitotic nuclear division	13	7.29 × 10^−8^	NCAPG2, AURKA, CDK1
**Molecular Function ** **(MFs)**	GO:0005524	ATP binding	30	3.80 × 10^−8^	TOP2A, AURKA, CDK1, MCM2
	GO:0005515	Protein binding	83	2.65 × 10^−7^	TOP2A, NCAPG2, BRCA1, MCM2, AURKA, CCNB1, CDK1
	GO:0003678	DNA helicase activity	6	3.77 × 10^−7^	MCM2
	GO:0003682	Chromatin binding	12	4.13 × 10^−5^	TOP2A, CDK1
	GO:0003677	DNA binding	25	1.20 × 10^−4^	TOP2A, BRCA1, MCM2
**Cellular Component**	GO:0005654	Nucleoplasm	56	4.91 × 10^−17^	TOP2A, NCAPG2, BRCA1, MCM2, AURKA, CCNB1, CDK1
	GO:0030496	Midbody	13	2.54 × 10^−11^	AURKA, CDK1
	GO:0042555	MCM complex	6	1.04 × 10^−9^	MCM2
	GO:0005634	Nucleus	65	2.18 × 10^−9^	TOP2A, NCAPG2, BRCA1, MCM2, AURKA, CCNB1, CDK1
	GO:0005819	Spindle	10	6.21 × 10^−8^	AURKA
	**hsa ID**	**Pathways**	**cDEGs** **(Counts)**	***p*-Value**	**Associated cHubGs**
**KEGG Pathway**	hsa03030	DNA replication	9	7.97 × 10^−11^	MCM2
	hsa04110	Cell cycle	12	5.37 × 10^−10^	CCNB1, CDK1, MCM2
	hsa04115	p53 signaling pathway	5	0.001158992	CCNB1, CDK1
	hsa04114	Oocyte meiosis	5	0.007240129	CCNB1, CDK1, AURKA
	hsa03460	Fanconianemia pathway	3	0.0485697	BRCA1

## Data Availability

Not applicable.

## Supplementary Materials

The following supporting information can be downloaded at: https://www.mdpi.com/article/10.3390/ijms23073968/s1.

## Abbreviations

CC	Cervical Cancer
CSCC	Cervical Squamous Cell Carcinoma
HPV	Human Papillomavirus
(MLICs)	Middle- and Low-Income Countries
LIMMA	Linear Models for Microarray Data
PPI	Protein–Protein Interaction
ENCODE	Encyclopedia Of DNA Elements
MCODE	Molecular Complex Detection
DEGs	Differentially Expressed Genes
cDEGs	Common Differentially Expressed Genes
cHubGs	Common Hub Genes
cHubPs	Common Hub Proteins
KGs	Key Genes
KPs	Key Proteins
DR	Drug Repurposing
GO	Gene Ontology
KEGG	Kyoto Encyclopedia of Genes and Genomes
TFs	Transcription Factors
miRNAs	Micro-RNAs
MD	Molecular Dynamic
MM-PBSA	Molecular Mechanics Poisson–Boltzmann Surface Area
RMSD	Root Mean Square Deviation
3D	Three-Dimensional
PDB	Protein Data Bank
PLIP	Protein–Ligand Interaction Profiler
YASARA	Yet Another Scientific Artificial Reality Application
