# Supplementary material for: Bioinformatics Screening of Potential Biomarkers from mRNA Expression Profiles to Discover Drug Targets and Agents for Cervical Cancer
Source: Int J Mol Sci. 2022 Apr 2;23(7):3968. doi: 10.3390/ijms23073968 (PMC8999699; doi:10.3390/ijms23073968)
Supplement: Supplementary file 1 [file ijms-23-03968-s001.zip › ijms-1604272-supplementary.pdf]

**Table-S1:** 80 meta-drug agents

| Candidate drug agents Name | Reference |
|---|---|
| 5_Fluorouracil, Adriamycin, Bleomycin, Bleomycin_Sulfate, Brivanib,Celecoxib, Cetuximab, Chlorambucil, Ciclopirox, Curcumin, Cyclophosphamide, Decitabine, Docetaxel, Doxil, Doxorubicin, Eribulin_Mesylate, Erlotinib, toposide, Everolimus, Exatecan, Fluorouracil, Fucoxanthin, Gefitinib, Geldanamycin, Gemcitabine, Hycamtin, Ifosfamide, Imatinib, Irinotecan, LY900009, Lapatinib, MK1775, Melphalan, Mesna, Methotrexate, Mitolactol, Mitomycin_C, Nelfinavir, Olaparib, Paclitaxel, Pazopanib, Piperlongumine, Rucaparib, Sizofiran, Sorafenib, Sunitinib, TNP_470, Temsirolimus, Thioridazine, Tirapazamine, Topotecan, Topotecan_Hydrochloride, Trastuzumab, Triapine, Veliparib, Vincristine, Vinorelbine, Vorinostat, amifostine, apigenin, belotecan, bendamustine, cabazitaxel, camptothecin, carboplatin, chrysin, cisplatin, gimeracil, hydralazine, hydroxyurea, pemetrexed, pralatrexate, resveratrol, tegafur, thioguanosine, thrombospondin_1, trichostatin_A, valproate, vedotin | [1–24] |

**Table S2:** Different key protein lists for CC infection published by different research articles in different international reputed journals

| Author | Hub Genes | Common hub genes with at least 5 articles | Common hub genes with at least 6 articles |
|---|---|---|---|
| (Venkataramnan and Binti Zainol Izam Khan 2020)[25] | AURKA, UBE2C, TPX2, MCM5, GINS2 | | |
| (Y. Mei et al. 2020)[14] | APOBEC3B, DSG2, CXCL8, ABCA8, PLAGL1 | | |
| (Jinhui Liu et al. 2019)[15] | EPHX2, RMI2 | | |
| (Qiu et al. 2020)[16] | RRM2, CDC45, GINS2, HELLS, KNTC1, MCM2, MYBL2, PCNA, RAD54 L, RFC4, RFC5, TK1, TOP2A, TYMS | | |
| (Meneur et al. 2021)[17] | FASN, HK2, ACACB, PIF1, COX7A1, CKMT2, CPT1B, PDK4, DNA2, and COX4I2 | | |
| (X. Wu et al. 2019)[26] | TSPO, CCND1, FOS, CDK1, TOP2A, CCNB1, PCNA, BIRC5, MAD2L1 | | |
| (J. Mei et al. 2020)[27] | RIPOR2, CXCL8 | | |
| (Yi et al. 2020)[28] | CDK1, CCNB1, ITGB1, FN1, MMP9, STAT1 | | |
| (Deng, Zhu, and Huang 2016)[29] | GPS1, CCDC65, SLC9A7, VARS, SNX3, ENPP4, RGS7, LOC125943, LOC143903, LOC166602, BICC1, LOC253956, LOC220362, LOC206117, ADA, NAV1, LEF1 | | |
| (H. ju Yang et al. 2020)[30] | TYMS, MCM2, HELLS, TOP2A, CXCL8 | | |
| (J. Wang et al. 2020)[31] | DSG2, MMP1, SPP1, MCM2 | | |
| (Jinhui Liu, Yang, et al. 2020)[32] | EZH2, FLT1, GAPDH | | |
| (Ouyang et al. 2020)[33] | SNRPA, CCDC12 | | |
| (H. Chen et al. 2020)[34] | KNTC1 | AURKA, PCNA, CCNB1, CDC45, MCM2, TOP2A, CDK1 | CCNB1, CDC45, MCM2 TOP2A, CDK1 |
| (H. Xue et al. 2021)[35] | CDC45, GINS2, MCM2, PCNA | | |
| (Zhao et al. 2020)[36] | CDC6, CDK1, CDC45, BUB1, TOP2A, MCM4, CCNB2, CCNB1 | | |
| (Ma et al. 2020)[37] | ESR1, EPB41L3, EDNRB, ID4, PLAC8 | | |
| (Mallik et al. 2020)[38] | PAIP2, GRWD1, VPS4B, CRADD, LLPH, MRPL35, FAM177A1, STAT4, ASPSCR1, FABP7 | | |
| (Jinhui Liu, Li, et al. 2020)[39] | ACKR1 | | |
| (Tu et al. 2021)[40] | RFC5, POLE3, RAD51, RMI1, PALB2, HDAC1, MCM4, ESR1, FOS, E2F1 | | |
| (K. Wu et al. 2018)[41] | PCNA, CDK2, VEGFA, PIK3CA | | |
| (Jinhui Liu, Wu, et al. 2020)[42] | CD1C, CD6 | | |
| (Jiamei Liu, Liu, and Yang 2021)[43] | NDC80, TIPIN, MCM3, MCM6, POLA1, PRC1 | | |
| (Z. Xu et al. 2017)[44] | CDK1, TOP2A, MCM2, AURKA, KIF20A, ESR1 | | |
| (Y. Liu et al. 2019)[45] | CCNA2, CDK1, CCND1, FGF2, IGF1, BCL2, VEGFA | | |
| (J. M. Xue et al. 2020)[46] | TOP2A, ECT2, RRM2, ANLN, NEK2, ASPM, BUB1B, CDK1, DTL, PRC1 | | |
| (M. Wang et al. 2018)[47] | MCM2, MMP2, COL1A1, JUN | | |
| (Yuan et al. 2020)[48] | TOP2A, AURKA, CHEK1, KIF11, MCM4, MKI67, DTL, FOXM1, SMC4, FBXO5, | | |
| (Mousavi et al. 2020)[49] | CDK1, PLK1 | | |
| (Xi Zhang and Wang 2019)[50] | SMYD2, EGLN1, TNFRSF10D, FUT11, SYTL3, MMP8, EREG | | |

| | |
|---|---|
| (He et al. 2021)[51] | CDC45 |
| (Q. Chen et al. 2018)[52] | TP53, MMP1, NOTCH1, SMAD4, NFKB1 |
| (Shiyan Li et al. 2021)[53] | SLC25A5, ENO1, ANLN, RIBC2, PTTG1, MCM5 |
| (Fu, Wu, and Xue 2018)[54] | ARFGAP3 |
| (B. Wu and Xi 2021b)[55] | CDC45, ORC1, RPA1, CDT1, TARDBP, RBMX, SRSF3, SRSF1, RFC5, RFC2, MSH6, DTL, MSH2 |
| (Meng et al. 2020)[56] | DSG2, ITM2A, CENPM, RIBC2, MEIS2 |
| (Ding et al. 2020)[57] | PTK2, NRP1, PRKAA1, HMGCS1 |
| (Shufeng Li et al. 2020)[58] | CCNB1 |
| (B. Wu and Xi 2021a)[59] | NUSAP1, TOP2A, KIF2C, NDC80, ASPM, KIF20A, CDK1, KIF11, BIRC5, MCM2, CHEK1 |
| (Wen, Liu, and Cui 2020)[60] | BRCA1, CDCA8, ASPM, CDC45, RAD51, HMMR, CENPF, EXO1, DTL, ZWINT |
| (Suman and Mishra 2018)[61] | TOP2A, BIRC5, AURKA, CCNB2 |
| (Xue Zhang et al. 2020)[62] | ASPM, CDC45, CDC7, CENPO, PRIM2, RFC4, EXO1, TOP2A, CENPQ, RAD54L, ZWILCH, NCAPH, KIAA0101, TOPBP1 |
| (Sun et al. 2019)[63] | TYMS, SASH1, CDK1, AURKA |
| (Oany et al. 2021)[64] | PTPRC, ITGAM, IL10, TYROBP, ITGB2, CCR5, ITGAX, CSF1R, LILRB2, CXCR4, STAT3, and CYBB |
| (Xiao et al. 2021)[65] | RFC4, ATAD2, TRIP13, NUF2, FOXM1, ECT2, KIF14, CDK2, KNTC1, DNA2. |
| (Yu et al. 2018)[66] | IGF2R, DNAJC6, FZD4, CBL, CLTC, ARPC3, VAMP2, ITGB3, VAMP4, ITGB1, MAPK8, KIF2A, CLASP1, SMC3, STAG2, STAG1, H2AFZ, RBBP4, UBE2D3 |
| (Xiaoyu Zhang et al. 2019)[67] | JUN, DCN, THBS1, HLA-DRA, EDN1, TIMP3, TGFB2, OAS3, OASL, OAS1, PDGFB, MMP1, EGR1, SDC4, SERPINE1 |
| (F. Xu, Shen, and Xu 2021)[68] | CCR7, CD3D, CD3E, ITGB2, FAM133A, TP53 |
| (Jiang et al. 2021)[69] | APOD, CXCL8, MMP1, MMP3, PLOD2, PTGDS, SNX10, SPP1 |
| (C. Yang, Xu, and Jin 2016)[70] | PBXl, LAMC2 |
| (Luo et al. 2021)[71] | CDK1, BUB1, KIF11, NDC80, BUB1B, CCNB2, PCNA, CCNB1, MAD2L1 and CDCA8, |
| (Tong et al. 2021)[72] | RAD23A, PGD, ITPA, IDH1, THBS1 |
| (Z. Zhang et al. 2021)[73] | SELL, CX3CR1, FCGR2B, CD38, IL15, HLA-DRA, CCL8, CD79A, HLA-DMA, HLA-DPA1 |

**Table-S3**. List of 116 genes that were identified as upregulated or downregulated using the GSE6791 GSE27678, GSE63514, and GSE9750 microarray datasets.

| Type of DEGs | Differentially expressed genes (DEGs) |
|---|---|
| Downregulated DEGs | ENDOU, HOPX, CRNN, MAL, UPK1A, EMP1, EDN3, SLC27A6, KRT1, GYS2, ESR1, ALOX12, KRT2, HSPB8, EREG, KLK12, CRISP3, CFD, HPGD, PDGFD, GREB1, APOD, AR, KRT4, AQP1, SYNGR1, PEG3, TGFBR3, CYP2B6, NDN, ABCA8, SPON1, FAM107A, DACH1, SORBS1, IGFBP5, SLIT2, CYP2B7P |
| Upregulated DEGs | CDKN2A, CHAF1A, WHSC1, MCM2, NEK2, DTL, MCM5, GINS2, RAD54L, KIF18B, KNTC1, FOXM1, AURKA, DSG2, TYMS, MCM6, GTSE1, GINS1, UBE2C, ECT2, SPAG5, KIF4A, MCM7, KIF2C, KIF14, TIMELESS, TPX2, MELK, FANCG, RFC4, CENPI, PLOD2, CDC20, EZH2, TOP2A, CDK1, ATAD2, RRM2, BIRC5, LOC101928615, FNDC3B, ATP2C1, FEN1, FANCI, WDHD1, ZWINT, ATP13A3, CENPF, NEMP1, MCM4, SMC4, PRC1, GMNN, ASPM, DHFR, MCM3, RAD51AP1, POLQ, SMS, TOPBP1, KIF20A, GMPS, CENPN, NCAPG2, BRCA1, HJURP, DLGAP5, NUSAP1, TTK, CDC7, NCAPG, SHCBP1, HMMR, CKS2, RFC5, CCNB1, KRT17, JUP |

**Table-S4:** The top 20 significantly (p-value<0.001) enriched GO functions and KEGG pathways by cDEGs involving KGs with CC diseases.

| Biological Process | | | | |
|---|---|---|---|---|
| **Term** | **Count** | **PValue** | **cDEGs** | **FDR** |
| GO:0006260~DNA replication | 18 | 1.58E-16 | RFC5, GINS2, FEN1, RFC4, RRM2, MCM7, CDC7, BRCA1, CHAF1A, MCM3, TIMELESS, CDK1, MCM4, MCM5, TOPBP1, MCM6, DTL, MCM2 | 1.43E-13 |
| GO:0051301~cell division | 22 | 7.94E-15 | SPAG5, UBE2C, NCAPG2, KIF14, NCAPG, CDC7, SMC4, ZWINT, AURKA, CDC20, TPX2, CENPF, CCNB1, KIF18B, CKS2, TIMELESS, CDK1, BIRC5, KNTC1, MCM5, NEK2, KIF2C | 3.57E-12 |
| GO:0000082~G1/S transition of mitotic cell cycle | 12 | 5.61E-11 | DHFR, RRM2, MCM7, CDKN2A, CDK1, MCM3, MCM4, CDC7, MCM5, MCM6, TYMS, MCM2 | 1.68E-08 |
| GO:0006270~DNA replication initiation | 8 | 1.18E-09 | MCM7, MCM3, MCM4, CDC7, MCM5, TOPBP1, MCM6, MCM2 | 2.65E-07 |
| GO:0007067~mitotic nuclear division | 13 | 7.29E-08 | NCAPG2, AURKA, CDC20, ASPM, TPX2, CENPF, TIMELESS, CDK1, BIRC5, KNTC1, NEK2, KIF2C, CENPN | 1.31E-05 |
| GO:0006268~DNA unwinding involved in DNA replication | 5 | 3.42E-07 | TOP2A, MCM7, MCM4, MCM6, MCM2 | 4.72E-05 |
| GO:0006281~DNA repair | 12 | 3.67E-07 | RFC5, RAD51AP1, FANCI, POLQ, FEN1, CHAF1A, CDK1, RAD54L, BRCA1, TOPBP1, FOXM1, FANCG | 4.72E-05 |
| GO:0000086~G2/M transition of mitotic cell cycle | 9 | 3.04E-06 | TPX2, CCNB1, MELK, CDK1, BIRC5, NEK2, HMMR, FOXM1, AURKA | 3.04E-04 |
| GO:0007059~chromosome segregation | 7 | 5.14E-06 | TOP2A, CENPF, SPAG5, HJURP, CENPN, NEK2, BRCA1 | 4.21E-04 |
| GO:0008283~cell proliferation | 12 | 2.58E-05 | TPX2, AR, CENPF, DACH1, MELK, MCM7, CDK1, CKS2, EMP1, KIF2C, TYMS, DLGAP5 | 0.001938 |
| GO:0007051~spindle organization | 4 | 1.40E-04 | ASPM, SPAG5, TTK, AURKA | 0.009699 |
| GO:0048146~positive regulation of fibroblast proliferation | 5 | 4.14E-04 | CCNB1, PDGFD, ESR1, EREG, AQP1 | 0.021432 |
| GO:0006974~cellular response to DNA damage stimulus | 8 | 4.15E-04 | TOP2A, POLQ, MCM7, TIMELESS, BRCA1, TOPBP1, DTL, FANCG | 0.021432 |
| GO:0051439~regulation of ubiquitin-protein ligase activity involved in mitotic cell cycle | 4 | 4.29E-04 | CDC20, CCNB1, UBE2C, CDK1 | 0.021432 |
| GO:0000083~regulation of transcription involved in G1/S transition of mitotic cell cycle | 4 | 4.29E-04 | DHFR, RRM2, CDK1, TYMS | 0.021432 |
| GO:0070301~cellular response to hydrogen peroxide | 5 | 5.09E-04 | PDGFD, CDK1, ECT2, EZH2, AQP1 | 0.024028 |
| GO:0007049~cell cycle | 8 | 5.34E-04 | CDC20, CHAF1A, GMNN, HJURP, BRCA1, FOXM1, AURKA, MCM2 | 0.024028 |

| | | | | |
|---|---|---|---|---|
| GO:0045840~positive regulation of mitotic nuclear division | 4 | 6.20E-04 | EDN3, NUSAP1, EREG, AURKA | 0.026591 |
| GO:1901796~regulation of signal transduction by p53 class mediator | 6 | 0.001283 | RFC5, TPX2, RFC4, BRCA1, TOPBP1, AURKA | 0.05019 |
| GO:0000724~double-strand break repair via homologous recombination | 5 | 0.001363 | RAD51AP1, POLQ, FEN1, RAD54L, BRCA1 | 0.051125 |

**Molecular Function**

| | | | | |
|---|---|---|---|---|
| GO:0005524~ATP binding | 30 | 3.80E-08 | TOP2A, MCM7, GMPS, KIF14, TTK, ATP2C1, SMC4, AURKA, RAD54L, NEK2, RFC5, POLQ, RFC4, UBE2C, ATAD2, CDC7, ABCA8, TPX2, KIF18B, MELK, KIF4A, MCM3, CDK1, MCM4, MCM5, KIF2C, KIF20A, MCM6, ATP13A3, MCM2 | 9.69E-06 |
| GO:0005515~protein binding | 83 | 2.65E-07 | TOP2A, SPON1, FEN1, MCM7, HSPB8, NCAPG2, GMNN, HJURP, KIF14, BRCA1, FOXM1, SMC4, AQP1, CDC20, CHAF1A, DACH1, NUSAP1, KNTC1, NEK2, WHSC1, TOPBP1, GTSE1, DLGAP5, RFC5, WDHD1, RFC4, KRT4, IGFBP5, KRT2, KRT1, EMP1, EREG, TGFBR3, AR, MELK, CKS2, MCM3, TIMELESS, MAL, BIRC5, MCM4, MCM5, KIF2C, KIF20A, MCM6, DTL, MCM2, NCAPG, TTK, ALOX12, HMMR, AURKA, RAD51AP1, SYNGR1, CCNB1, RAD54L, APOD, SLIT2, ECT2, FANCI, GINS2, POLQ, RRM2, JUP, SPAG5, CDKN2A, UBE2C, CDC7, SORBS1, ESR1, SHCBP1, FANCG, HOPX, ZWINT, TPX2, CENPF, KIF18B, KRT17, CENPI, PRC1, KIF4A, CDK1, EZH2 | 3.20E-05 |
| GO:0003678~DNA helicase activity | 6 | 3.77E-07 | MCM7, MCM3, MCM4, MCM5, MCM6, MCM2 | 3.20E-05 |
| GO:0003682~chromatin binding | 12 | 4.13E-05 | TOP2A, POLQ, AR, CENPF, CHAF1A, ATAD2, CDK1, CKS2, MCM5, WHSC1, ESR1, EZH2 | 0.002632 |
| GO:0003677~DNA binding | 25 | 1.20E-04 | TOP2A, FEN1, MCM7, HJURP, BRCA1, FOXM1, DACH1, NUSAP1, RAD54L, TOPBP1, RFC5, FANCI, WDHD1, POLQ, RFC4, CDKN2A, ESR1, HOPX, AR, KIF4A, MCM3, MCM4, MCM6, MCM2, EZH2 | 0.006144 |
| GO:0019901~protein kinase binding | 10 | 6.90E-04 | TPX2, CCNB1, JUP, CDKN2A, PRC1, KIF14, CKS2, KIF20A, FOXM1, AURKA | 0.029308 |
| GO:0019899~enzyme binding | 9 | 0.001342 | RFC5, TOP2A, CDC20, AR, RFC4, BIRC5, BRCA1, ESR1, MCM2 | 0.048903 |
| GO:0003777~microtubule motor activity | 5 | 0.001725 | KIF18B, KIF4A, KIF14, KIF2C, KIF20A | 0.050003 |
| GO:0043142~single-stranded DNA-dependent ATPase activity | 3 | 0.001765 | RFC5, POLQ, RFC4 | 0.050003 |
| GO:0008017~microtubule binding | 7 | 0.002198 | KIF18B, PRC1, KIF4A, NUSAP1, KIF14, BIRC5, KIF20A | 0.056039 |
| GO:0008574~ATP-dependent microtubule motor activity, plus-end-directed | 3 | 0.00518 | KIF18B, KIF4A, KIF14 | 0.120084 |
| GO:0016887~ATPase activity | 6 | 0.006431 | ATAD2, KIF14, KIF2C, KIF20A, ATP13A3, ABCA8 | 0.136663 |
| GO:0042803~protein homodimerization activity | 12 | 0.007021 | TOP2A, GYS2, CENPF, UPK1A, JUP, HPGD, TIMELESS, BIRC5, TTK, SLIT2, TYMS, ECT2 | 0.137719 |

| | | | | |
|---|---|---|---|---|
| GO:0003684~damaged DNA binding | 4 | 0.007656 | POLQ, FEN1, BRCA1, FANCG | 0.139456 |
| GO:0004003~ATP-dependent DNA helicase activity | 3 | 0.017752 | MCM7, MCM4, MCM6 | 0.301781 |
| GO:0003697~single-stranded DNA binding | 4 | 0.021808 | RAD51AP1, MCM7, MCM4, MCM6 | 0.347565 |
| GO:0035173~histone kinase activity | 2 | 0.025349 | CCNB1, CDK1 | 0.380229 |
| GO:0015631~tubulin binding | 3 | 0.02957 | KIF14, BIRC5, BRCA1 | 0.41891 |
| GO:0086083~cell adhesive protein binding involved in bundle of His cell-Purkinje myocyte communication | 2 | 0.037783 | JUP, DSG2 | 0.507089 |
| GO:0042393~histone binding | 4 | 0.043499 | ATAD2, HJURP, CKS2, MCM2 | 0.533573 |

**Cellular Component**

| | | | | |
|---|---|---|---|---|
| GO:0005654~nucleoplasm | 56 | 4.91E-17 | TOP2A, FEN1, MCM7, HSPB8, NCAPG2, GMNN, HJURP, BRCA1, FOXM1, SMC4, CDC20, WHSC1, TOPBP1, GTSE1, RFC5, WDHD1, RFC4, PEG3, AR, MCM3, TIMELESS, BIRC5, MCM4, MCM5, KIF20A, MCM6, DTL, MCM2, HPGD, TYMS, AURKA, RAD51AP1, CCNB1, RAD54L, FANCI, GINS1, GINS2, POLQ, RRM2, SPAG5, CDKN2A, UBE2C, ATAD2, CDC7, SORBS1, ESR1, FANCG, DHFR, TPX2, CENPF, CENPI, PRC1, KIF4A, CDK1, CENPN, EZH2 | 7.55E-15 |
| GO:0030496~midbody | 13 | 2.54E-11 | SPAG5, KIF14, SHCBP1, AURKA, ASPM, CENPF, PRC1, KIF4A, CDK1, BIRC5, NEK2, KIF20A, ECT2 | 1.95E-09 |
| GO:0042555~MCM complex | 6 | 1.04E-09 | MCM7, MCM3, MCM4, MCM5, MCM6, MCM2 | 5.32E-08 |
| GO:0005634~nucleus | 65 | 2.18E-09 | TOP2A, FEN1, MCM7, HSPB8, NCAPG2, GMNN, FAM107A, HJURP, KIF14, BRCA1, FOXM1, SMC4, AQP1, CDC20, CHAF1A, DACH1, NUSAP1, KNTC1, NEK2, WHSC1, TOPBP1, DLGAP5, KRT4, PEG3, KRT2, KRT1, ASPM, AR, MELK, MCM3, TIMELESS, BIRC5, MCM4, MCM5, KIF2C, MCM6, DTL, MCM2, NCAPG, TYMS, AURKA, RAD51AP1, CCNB1, NDN, RAD54L, ECT2, GINS1, GINS2, RRM2, JUP, SPAG5, CDKN2A, ATAD2, CDC7, SORBS1, ESR1, HOPX, ZWINT, TPX2, CENPF, KIF18B, CENPI, PRC1, CDK1, EZH2 | 8.38E-08 |
| GO:0005819~spindle | 10 | 6.21E-08 | CDC20, TPX2, CENPF, PRC1, NUSAP1, BIRC5, TTK, KIF20A, SHCBP1, AURKA | 1.91E-06 |
| GO:0005876~spindle microtubule | 7 | 2.90E-07 | SPAG5, PRC1, KIF4A, NUSAP1, CDK1, BIRC5, AURKA | 6.74E-06 |
| GO:0005737~cytoplasm | 59 | 3.07E-07 | TOP2A, MCM7, HSPB8, GMNN, HJURP, BRCA1, FOXM1, SMC4, AQP1, GYS2, CDC20, ENDOU, DACH1, NUSAP1, KNTC1, NEK2, WHSC1, TOPBP1, DLGAP5, WDHD1, KRT2, TGFBR3, ASPM, AR, BIRC5, DTL, MCM2, HPGD, GMPS, NCAPG, TTK, ALOX12, TYMS, CCNB1, SLIT2, ECT2, FANCI, GINS1, RRM2, JUP, SPAG5, CDKN2A, UBE2C, CDC7, SORBS1, ESR1, SHCBP1, FANCG, HOPX, ZWINT, CRNN, CENPF, KIF18B, KRT17, CENPI, PRC1, KIF4A, CDK1, EZH2 | 6.74E-06 |

| | | | | |
|---|---|---|---|---|
| GO:0000922~spindle pole | 9 | 3.84E-07 | CDC20, TPX2, CENPF, CCNB1, SPAG5, PRC1, KNTC1, NEK2, TOPBP1 | 7.40E-06 |
| GO:0000777~condensed chromosome kinetochore | 8 | 1.13E-06 | SPAG5, HJURP, BIRC5, KNTC1, KIF2C, CENPN, NEK2, ZWINT | 1.94E-05 |
| GO:0005694~chromosome | 8 | 4.03E-06 | POLQ, KIF4A, NUSAP1, BRCA1, TOPBP1, WHSC1, SMC4, DTL | 6.21E-05 |
| GO:0000776~kinetochore | 7 | 1.11E-05 | CENPF, SPAG5, CENPI, TTK, KIF2C, NEK2, ZWINT | 1.55E-04 |
| GO:0000784~nuclear chromosome, telomeric region | 8 | 1.65E-05 | FEN1, MCM7, CDK1, MCM3, MCM4, MCM5, MCM6, MCM2 | 2.12E-04 |
| GO:0005813~centrosome | 12 | 6.86E-05 | CDC20, CENPF, CCNB1, CDK1, MCM3, NDN, NCAPG, NEK2, TOPBP1, SORBS1, DTL, AURKA | 8.12E-04 |
| GO:0005874~microtubule | 10 | 1.38E-04 | TPX2, KIF18B, KIF4A, NUSAP1, KIF14, BIRC5, KIF2C, NEK2, KIF20A, AURKA | 0.001519 |
| GO:0015630~microtubule cytoskeleton | 7 | 2.11E-04 | TPX2, PRC1, TIMELESS, KIF2C, CDC7, AURKA, MCM2 | 0.002171 |
| GO:0005829~cytosol | 37 | 3.13E-04 | MCM7, HPGD, GMNN, GMPS, KIF14, NCAPG, ALOX12, HMMR, TYMS, SMC4, AURKA, CDC20, GYS2, CCNB1, NDN, KNTC1, NEK2, ECT2, GTSE1, RRM2, JUP, CDKN2A, UBE2C, SORBS1, ZWINT, DHFR, AR, TPX2, CENPF, PRC1, CENPI, KIF4A, SMS, CDK1, BIRC5, CENPN, KIF2C | 0.002941 |
| GO:0005871~kinesin complex | 5 | 3.25E-04 | KIF18B, KIF4A, KIF14, KIF2C, KIF20A | 0.002941 |
| GO:0045120~pronucleus | 3 | 7.84E-04 | CENPF, EZH2, AURKA | 0.006356 |
| GO:0016020~membrane | 26 | 0.001825 | FEN1, MCM7, NCAPG2, KIF14, NCAPG, TTK, ALOX12, HMMR, ATP2C1, CCNB1, SLIT2, GTSE1, FANCI, KRT2, KRT1, EMP1, ESR1, CRNN, MELK, KIF4A, MCM3, CDK1, MCM4, MCM5, KIF2C, ATP13A3 | 0.014051 |
| GO:0072686~mitotic spindle | 4 | 0.002084 | SPAG5, CDK1, ECT2, AURKA | 0.015286 |

**KEGG Pathway**

| Term | Count | PValue | cDEGs |
|---|---|---|---|
| DNA replication(hsa03030) | 9 | 7.97E-11 | RFC5, FEN1, RFC4, MCM7, MCM3, MCM4, MCM5, MCM6, MCM2 |
| Cell cycle (hsa04110) | 12 | 5.37E-10 | CDC20, CCNB1, MCM7, CDKN2A, CDK1, MCM3, MCM4, TTK, CDC7, MCM5, MCM6, MCM2 |
| p53 signaling pathway (hsa04115) | 5 | 0.001159 | CCNB1, RRM2, CDKN2A, CDK1, GTSE1 |
| Oocyte meiosis (hsa04114) | 5 | 0.00724 | CDC20, AR, CCNB1, CDK1, AURKA |
| Fanconi anemia pathway (hsa03460:) | 3 | 0.04857 | FANCI, BRCA1, FANCG |

## Reference:


1. Marquina, G.; Manzano, A.; Casado, A. Targeted Agents in Cervical Cancer: Beyond Bevacizumab. *Curr. Oncol. Rep.* **2018**, *20*, doi:10.1007/s11912-018-0680-3.

2. Sharma, S.; Deep, A.; Sharma, A.K. Current Treatment for Cervical Cancer: An Update. *Anticancer. Agents Med. Chem.* **2020**, *20*, doi:10.2174/1871520620666200224093301.

3. Verschraegen, C.F. Irinotecan for the treatment of cervical cancer. *Oncology (Williston Park).* 2002, *16*.

4. Su, J.; Zhang, F.; Li, X.; Liu, Z. Osthole promotes the suppressive effects of cisplatin on NRF2 expression to prevent drug-resistant cervical cancer progression. *Biochem. Biophys. Res. Commun.* **2019**, *514*, doi:10.1016/j.bbrc.2019.04.021.

5. Ackermann, S.; Beckmann, M.W.; Thiel, F.; Bogenrieder, T. Topotecan in cervical cancer. *Int. J. Gynecol. Cancer* 2007, *17*.

6. Chandimali, N.; Sun, H.N.; Park, Y.H.; Kwon, T. BRM270 suppresses cervical cancer stem cell characteristics and progression by inhibiting SOX2. *In Vivo (Brooklyn).* **2020**, *34*, doi:10.21873/invivo.11879.

7. Markman, M. Advances in cervical cancer pharmacotherapies. *Expert Rev. Clin. Pharmacol.* 2014, *7*.

8. Moga, M.A.; Dima, L.; Balan, A.; Blidaru, A.; Dimienescu, O.G.; Podasca, C.; Toma, S. Are bioactive molecules from seaweeds a novel and challenging option for the prevention of HPV infection and cervical cancer therapy?—a review. *Int. J. Mol. Sci.* 2021, *22*.

9. Lee, S.W.; Kim, Y.M.; Kim, M.B.; Kim, D.Y.; Kim, J.H.; Nam, J.H.; Kim, Y.T. Chemosensitivity of uterine cervical cancer demonstrated by the histoculture drug response assay. *Tohoku J. Exp. Med.* **2009**, *219*, doi:10.1620/tjem.219.277.

10. Small, W. Potential for use of amifostine in cervical cancer. *Semin. Oncol.* **2002**, *29*, doi:10.1053/sonc.2002.37366.

11. Ai, Z.; Wang, J.; Xu, Y.; Teng, Y. Bioinformatics analysis reveals potential candidate drugs for cervical cancer. *J. Obstet. Gynaecol. Res.* **2013**, *39*, doi:10.1111/jog.12022.

12. Ujhelyi, Z.; Kalantari, A.; Vecsernyés, M.; Róka, E.; Fenyvesi, F.; Póka, R.; Kozma, B.; Bácskay, I. The enhanced inhibitory effect of different antitumor agents in self-microemulsifying drug delivery systems on human cervical cancer HeLa cells. *Molecules* **2015**, *20*, doi:10.3390/molecules200713226.

13. Duenas-Gonzalez, A.; Gonzalez-Fierro, A. Pharmacodynamics of current and emerging treatments for cervical cancer. *Expert Opin. Drug Metab. Toxicol.* **2019**, *15*, doi:10.1080/17425255.2019.1648431.

14. Mei, Y.; Jiang, P.; Shen, N.; Fu, S.; Zhang, J. Identification of miRNA-mRNA Regulatory Network and Construction of Prognostic Signature in Cervical Cancer. *DNA Cell Biol.* **2020**, *39*, doi:10.1089/dna.2020.5452.

15. Liu, J.; Nie, S.; Gao, M.; Jiang, Y.; Wan, Y.; Ma, X.; Zhou, S.; Cheng, W. Identification of EPHX2 and RMI2 as two novel key genes in cervical squamous cell carcinoma by an integrated bioinformatic analysis. *J. Cell. Physiol.* **2019**, *234*, doi:10.1002/jcp.28731.

16. Qiu, H.Z.; Huang, J.; Xiang, C.C.; Li, R.; Zuo, E.D.; Zhang, Y.; Shan, L.; Cheng, X. Screening and Discovery of New Potential Biomarkers and Small Molecule Drugs for Cervical Cancer: A Bioinformatics Analysis. *Technol. Cancer Res. Treat.* **2020**, *19*, doi:10.1177/1533033820980112.

17. Meneur, C.; Eswaran, S.; Adiga, D.; Sriharikrishnaa, S.; Nadeem, K.G.; Mallya, S.; Chakrabarty, S.; Kabekkodu, S.P. Analysis of Nuclear Encoded Mitochondrial Gene Networks in Cervical Cancer. *Asian Pacific J. Cancer Prev.* **2021**, *22*, doi:10.31557/APJCP.2021.22.6.1799.

18. Barra, F.; Lorusso, D.; Leone Roberti Maggiore, U.; Ditto, A.; Bogani, G.; Raspagliesi, F.; Ferrero, S. Investigational drugs for the treatment of cervical cancer. *Expert Opin. Investig. Drugs* 2017, *26*.

19. Serrano-Olvera, A.; Cetina, L.; Coronel, J.; Dueñas-González, A. Emerging drugs for the treatment of cervical cancer. *Expert Opin. Emerg. Drugs* 2015, *20*.

20. Kamura, T.; Ushijima, K. Chemotherapy for advanced or recurrent cervical cancer. *Taiwan. J. Obstet. Gynecol.* 2013, *52*.

21. Klopp, A.H.; Eifel, P.J. Chemoradiotherapy for cervical cancer in 2010. *Curr. Oncol. Rep.* **2011**, *13*, doi:10.1007/s11912-010-0134-z.

22. Tao, X.; Hu, W.; Ramirez, P.T.; Kavanagh, J.J. Chemotherapy for recurrent and metastatic cervical cancer. *Gynecol. Oncol.* **2008**, *110*, doi:10.1016/j.ygyno.2008.04.024.

23. Diaz-Padilla, I.; Monk, B.J.; Mackay, H.J.; Oaknin, A. Treatment of metastatic cervical cancer: Future directions involving targeted agents. *Crit. Rev. Oncol. Hematol.* 2013, *85*.

24. Tierney, J.F.; Vale, C.; Symonds, P. Concomitant and Neoadjuvant Chemotherapy for Cervical Cancer. *Clin. Oncol.* **2008**, *20*, doi:10.1016/j.clon.2008.04.003.

25. Venkataramnan, S.; Binti Zainol Izam Khan, W.N. CERVICAL CANCER AND GENE EXPRESSION ANALYSIS WITH KEY GENES IDENTIFICATION BY COMPUTATIONAL METHOD. *J. Bio Innov.* **2020**, *9*, doi:10.46344/jbino.2020.v09i05.26.

26. Wu, X.; Peng, L.; Zhang, Y.; Chen, S.; Lei, Q.; Li, G.; Zhang, C. Identification of key genes and pathways in cervical cancer by bioinformatics analysis. *Int. J. Med. Sci.* **2019**, *16*, doi:10.7150/ijms.34172.

27. Mei, J.; Xing, Y.; Lv, J.; Gu, D.; Pan, J.; Zhang, Y.; Liu, J. Construction of an immune-related gene signature for prediction of prognosis in patients with cervical cancer. *Int. Immunopharmacol.* **2020**, *88*, doi:10.1016/j.intimp.2020.106882.

28. Yi, Y.; Fang, Y.; Wu, K.; Liu, Y.; Zhang, W. Comprehensive gene and pathway analysis of cervical cancer progression. *Oncol. Lett.* **2020**, *19*, doi:10.3892/ol.2020.11439.

29. Deng, S.P.; Zhu, L.; Huang, D.S. Predicting Hub Genes Associated with Cervical Cancer through Gene Co-Expression Networks. *IEEE/ACM Trans. Comput. Biol. Bioinforma.* **2016**, *13*, doi:10.1109/TCBB.2015.2476790.

30. Yang, H. ju; Xue, J. min; Li, J.; Wan, L. hong; Zhu, Y. xi Identification of key genes and pathways of diagnosis and prognosis in cervical cancer by bioinformatics analysis. *Mol. Genet. Genomic Med.* **2020**, *8*, doi:10.1002/mgg3.1200.

31. Wang, J.; Zheng, H.; Han, Y.; Wang, G.; Li, Y. A Novel Four-Gene Prognostic Signature as a Risk Biomarker in Cervical Cancer. *Int. J. Genomics* **2020**, *2020*, doi:10.1155/2020/4535820.

32. Liu, J.; Yang, J.; Gao, F.; Li, S.; Nie, S.; Meng, H.; Sun, R.; Wan, Y.; Jiang, Y.; Ma, X.; et al. A microRNA-Messenger RNA Regulatory Network and Its Prognostic Value in Cervical Cancer. *DNA Cell Biol.* **2020**, *39*, doi:10.1089/dna.2020.5590.

33. Ouyang, D.; Ouyang, D.; Yang, P.; Cai, J.; Sun, S.; Wang, Z. Comprehensive analysis of prognostic alternative splicing signature in cervical cancer. *Cancer Cell Int.* **2020**, *20*, doi:10.1186/s12935-020-01299-4.

34. Chen, H.; Wang, X.; Jia, H.; Tao, Y.; Zhou, H.; Wang, M.; Wang, X.; Fang, X. Bioinformatics analysis of key genes and pathways of cervical cancer. *Onco. Targets. Ther.* **2020**, *13*, 13275–13283, doi:10.2147/OTT.S281533.

35. Xue, H.; Sun, Z.; Wu, W.; Du, D.; Liao, S. Identification of hub genes as potential prognostic biomarkers in cervical cancer using comprehensive bioinformatics analysis and validation studies. *Cancer Manag. Res.* **2021**, *13*, doi:10.2147/CMAR.S282989.

36. Zhao, Q.; Li, H.; Zhu, L.; Hu, S.; Xi, X.; Liu, Y.; Liu, J.; Zhong, T. Bioinformatics analysis shows that top2a functions as a key candidate gene in the progression of cervical cancer. *Biomed. Reports* **2020**, *13*, doi:10.3892/br.2020.1328.

37. Ma, X.; Liu, J.; Wang, H.; Jiang, Y.; Wan, Y.; Xia, Y.; Cheng, W. Identification of crucial aberrantly

methylated and differentially expressed genes related to cervical cancer using an integrated bioinformatics analysis. *Biosci. Rep.* **2020**, *40*, doi:10.1042/BSR20194365.

38. Mallik, S.; Seth, S.; Bhadra, T.; Zhao, Z. A linear regression and deep learning approach for detecting reliable genetic alterations in cancer using dna methylation and gene expression data. *Genes (Basel).* **2020**, *11*, doi:10.3390/genes11080931.

39. Liu, J.; Li, S.; Lin, L.; Jiang, Y.; Wan, Y.; Zhou, S.; Cheng, W. Co-expression network analysis identified atypical chemokine receptor 1 (ACKR1) association with lymph node metastasis and prognosis in cervical cancer. *Cancer Biomarkers* **2020**, *27*, doi:10.3233/CBM-190533.

40. Tu, S.; Zhang, H.; Yang, X.; Wen, W.; Song, K.; Yu, X.; Qu, X. Screening of cervical cancer-related hub genes based on comprehensive bioinformatics analysis. *Cancer Biomarkers* **2021**, doi:10.3233/cbm-203262.

41. Wu, K.; Yi, Y.; Liu, F.; Wu, W.; Chen, Y.; Zhang, W. Identification of key pathways and genes in the progression of cervical cancer using bioinformatics analysis. *Oncol. Lett.* **2018**, *16*, doi:10.3892/ol.2018.8768.

42. Liu, J.; Wu, Z.; Wang, Y.; Nie, S.; Sun, R.; Yang, J.; Cheng, W. A prognostic signature based on immune-related genes for cervical squamous cell carcinoma and endocervical adenocarcinoma. *Int. Immunopharmacol.* **2020**, *88*, doi:10.1016/j.intimp.2020.106884.

43. Liu, J.; Liu, S.; Yang, X. Construction of Gene Modules and Analysis of Prognostic Biomarkers for Cervical Cancer by Weighted Gene Co-Expression Network Analysis. *Front. Oncol.* **2021**, *11*, doi:10.3389/fonc.2021.542063.

44. Xu, Z.; Zhou, Y.; Shi, F.; Cao, Y.; Dinh, T.L.A.; Wan, J.; Zhao, M. Investigation of differentially-expressed microRNAs and genes in cervical cancer using an integrated bioinformatics analysis. *Oncol. Lett.* **2017**, *13*, doi:10.3892/ol.2017.5766.

45. Liu, Y.; Yi, Y.; Wu, W.; Wu, K.; Zhang, W. Bioinformatics prediction and analysis of hub genes and pathways of three types of gynecological cancer. *Oncol. Lett.* **2019**, *18*, doi:10.3892/ol.2019.10371.

46. Xue, J.M.; Liu, Y.; Wan, L.H.; Zhu, Y.X. Comprehensive analysis of differential gene expression to identify common gene signatures in multiple cancers. *Med. Sci. Monit.* **2020**, *26*, doi:10.12659/MSM.919953.

47. Wang, M.; Li, L.; Liu, J.; Wang, J. A gene interaction network-based method to measure the common and heterogeneous mechanisms of gynecological cancer. *Mol. Med. Rep.* **2018**, *18*, doi:10.3892/mmr.2018.8961.

48. Yuan, Y.; Shi, X.; Li, B.; Peng, M.; Zhu, T.; Lv, G.; Liu, L.; Jin, H.; Li, L.; Qin, D. Integrated analysis of key microRNAs /TFs /mRNAs/ in HPV-positive cervical cancer based on microRNA sequencing and bioinformatics analysis. *Pathol. Res. Pract.* **2020**, *216*, doi:10.1016/j.prp.2020.152952.

49. Mousavi, S.Z.; Poortahmasebi, V.; Mokhtari-Azad, T.; Shahmahmoodi, S.; Farahmand, M.; Farzanehpour, M.; Jalilvand, S. The dysregulation of microarray gene expression in cervical cancer is associated with overexpression of a unique messenger rna signature. *Iran. J. Microbiol.* **2020**, *12*, doi:10.18502/ijm.v12i6.5039.

50. Zhang, X.; Wang, Y. Identification of hub genes and key pathways associated with the progression of gynecological cancer. *Oncol. Lett.* **2019**, *18*, doi:10.3892/ol.2019.11004.

51. He, Z.; Wang, X.; Yang, Z.; Jiang, Y.; Li, L.; Wang, X.; Song, Z.; Wang, X.; Wan, J.; Jiang, S.; et al. Expression and prognosis of CDC45 in cervical cancer based on the GEO database. *PeerJ* **2021**, *9*, doi:10.7717/peerj.12114.

52. Chen, Q.; Zeng, X.; Huang, D.; Qiu, X. Identification of differentially expressed miRNAs in early-stage cervical cancer with lymph node metastasis across the cancer genome atlas datasets. *Cancer Manag. Res.* **2018**, *10*, doi:10.2147/CMAR.S183488.

53. Li, S.; Han, F.; Qi, N.; Wen, L.; Li, J.; Feng, C.; Wang, Q. Determination of a six-gene prognostic model for cervical cancer based on WGCNA combined with LASSO and Cox-PH analysis. *World J. Surg. Oncol.* **2021**, *19*, 1–11, doi:10.1186/s12957-021-02384-2.

54. Fu, X.H.; Wu, Y.F.; Xue, F. Probing pathway-related modules in invasive squamous cervical cancer based on topological centrality of network strategy. *J. Cancer Res. Ther.* **2018**, *14*, doi:10.4103/0973-1482.187352.

55. Wu, B.; Xi, S. Bioinformatics analysis of the transcriptional expression of minichromosome maintenance proteins as potential indicators of survival in patients with cervical cancer. *BMC Cancer* **2021**, *21*, doi:10.1186/s12885-021-08674-y.

56. Meng, H.; Liu, J.; Qiu, J.; Nie, S.; Jiang, Y.; Wan, Y.; Cheng, W. Identification of Key Genes in Association with Progression and Prognosis in Cervical Squamous Cell Carcinoma. *DNA Cell Biol.* **2020**, *39*, doi:10.1089/dna.2019.5202.

57. Ding, H.; Zhang, L.; Zhang, C.; Song, J.; Jiang, Y. Screening of Significant Biomarkers Related to Prognosis of Cervical Cancer and Functional Study Based on lncRNA-associated ceRNA Regulatory Network. *Comb. Chem. High Throughput Screen.* **2020**, *24*, doi:10.2174/1386207323999200729113028.

58. Li, S.; Liu, N.; Piao, J.; Meng, F.; Li, Y. Ccnb1 expedites the progression of cervical squamous cell carcinoma via the regulation by foxm1. *Onco. Targets. Ther.* **2020**, *13*, doi:10.2147/OTT.S279951.

59. Wu, B.; Xi, S. Bioinformatics analysis of differentially expressed genes and pathways in the development of cervical cancer. *BMC Cancer* **2021**, *21*, doi:10.1186/s12885-021-08412-4.

60. Wen, X.; Liu, S.; Cui, M. Effect of BRCA1 on the Concurrent Chemoradiotherapy Resistance of Cervical Squamous Cell Carcinoma Based on Transcriptome Sequencing Analysis. *Biomed Res. Int.* **2020**, *2020*, doi:10.1155/2020/3598417.

61. Suman, S.; Mishra, A. Network analysis revealed aurora kinase dysregulation in five gynecological types of cancer. *Oncol. Lett.* **2018**, *15*, doi:10.3892/ol.2017.7368.

62. Zhang, X.; Bai, J.; Yuan, C.; Long, L.; Zheng, Z.; Wang, Q.; Chen, F.; Zhou, Y. Bioinformatics analysis and identification of potential genes related to pathogenesis of cervical intraepithelial neoplasia. *J. Cancer* **2020**, *11*, doi:10.7150/jca.38211.

63. Sun, D.; Han, L.; Cao, R.; Wang, H.; Jiang, J.; Deng, Y.; Yu, X. Prediction of a miRNA-mRNA functional synergistic network for cervical squamous cell carcinoma. *FEBS Open Bio* **2019**, *9*, doi:10.1002/2211-5463.12747.

64. Oany, A.R.; Mia, M.; Pervin, T.; Alyami, S.A.; Moni, M.A. Integrative systems biology approaches to identify potential biomarkers and pathways of cervical cancer. *J. Pers. Med.* **2021**, *11*, doi:10.3390/jpm11050363.

65. Xiao, L.; Zhang, S.; Zheng, Q.; Zhang, S. Dysregulation of KIF14 regulates the cell cycle and predicts poor prognosis in cervical cancer: A study based on integrated approaches. *Brazilian J. Med. Biol. Res.* **2021**, *54*, 1–10, doi:10.1590/1414-431X2021e11363.

66. Yu, D.; Li, Y.; Ming, Z.; Wang, H.; Dong, Z.; Qiu, L.; Wang, T. Comprehensive circular RNA expression profile in radiation-treated HeLa cells and analysis of radioresistance-related circRNAs. *PeerJ* **2018**, *2018*, doi:10.7717/peerj.5011.

67. Zhang, X.; Yang, P.; Luo, X.; Su, C.; Chen, Y.; Zhao, L.; Wei, L.; Zeng, H.; Varghese, Z.; Moorhead, J.F.; et al. High olive oil diets enhance cervical tumour growth in mice: Transcriptome analysis for potential candidate genes and pathways. *Lipids Health Dis.* **2019**, *18*, doi:10.1186/s12944-019-1023-6.

68. Xu, F.; Shen, J.; Xu, S. Multi-Omics Data Analyses Construct a Six Immune-Related Genes Prognostic

Model for Cervical Cancer in Tumor Microenvironment. *Front. Genet.* **2021**, *12*, doi:10.3389/fgene.2021.663617.

69. Jiang, P.; Cao, Y.; Gao, F.; Sun, W.; Liu, J.; Ma, Z.; Xie, M.; Fu, S. SNX10 and PTGDS are associated with the progression and prognosis of cervical squamous cell carcinoma. *BMC Cancer* **2021**, *21*, doi:10.1186/s12885-021-08212-w.

70. Yang, C.; Xu, X.; Jin, H. Identification of potential miRNAs and candidate genes of cervical intraepithelial neoplasia by bioinformatic analysis. *Eur. J. Gynaecol. Oncol.* **2016**, *37*, doi:10.12892/ejgo3131.2016.

71. Luo, H.; Li, Y.; Zhao, Y.; Chang, J.; Zhang, X.; Zou, B.; Gao, L.; Wang, W. Comprehensive Analysis of circRNA Expression Profiles During Cervical Carcinogenesis. *Front. Oncol.* **2021**, *11*, 1–13, doi:10.3389/fonc.2021.676609.

72. Tong, Y.; Sun, P.; Yong, J.; Zhang, H.; Huang, Y.; Guo, Y.; Yu, J.; Zhou, S.; Wang, Y.; Wang, Y.; et al. Radiogenomic Analysis of Papillary Thyroid Carcinoma for Prediction of Cervical Lymph Node Metastasis: A Preliminary Study. *Front. Oncol.* **2021**, *11*, doi:10.3389/fonc.2021.682998.

73. Zhang, Z.; Zhao, S.; Wang, K.; Shang, M.; Chen, Z.; Yang, H.; Chen, Y.; Chen, B. Identification of biomarkers associated with cervical lymph node metastasis in papillary thyroid carcinoma: Evidence from an integrated bioinformatic analysis. *Clin. Hemorheol. Microcirc.* **2021**, *78*, doi:10.3233/CH-201074.
